# A data-driven approach to a chemotherapy recommendation model based on deep learning for patients with colorectal cancer in Korea

**DOI:** 10.1186/s12911-020-01265-0

**Published:** 2020-09-22

**Authors:** Jin-Hyeok Park, Jeong-Heum Baek, Sun Jin Sym, Kang Yoon Lee, Youngho Lee

**Affiliations:** 1 Department of IT Convergence Engineering, Gachon University, 1342, Seongnam-daero, Sujeong-gu, Seongnam-si, Gyeonggi-do 13120 Republic of Korea; 2 Division of Colon and Rectal Surgery, Department of Surgery, Gil Medical Center, Gachon University College of Medicine, Incheon, 21565 Republic of Korea; 3 Division of Medical Oncology, Department of Internal Medicine, Gil Medical Center, Gachon University College of Medicine, Incheon, 21565 Republic of Korea; 4 Department of Computer Engineering, Gachon University, 1342, Seongnam-daero, Sujeong-gu, Seongnam-si, Gyeonggi-do 13120 Republic of Korea

**Keywords:** Colorectal Cancer, Knowledge-based clinical decision support system (CDSS), Deep learning, Chemotherapy recommendation

## Abstract

**Background:**

Clinical Decision Support Systems (CDSSs) have recently attracted attention as a method for minimizing medical errors. Existing CDSSs are limited in that they do not reflect actual data. To overcome this limitation, we propose a CDSS based on deep learning.

**Methods:**

We propose the Colorectal Cancer Chemotherapy Recommender (C3R), which is a deep learning-based chemotherapy recommendation model. Our model improves on existing CDSSs in which data-based decision making is not well supported. C3R is configured to study the clinical data collected at the Gachon Gil Medical Center and to recommend appropriate chemotherapy based on the data. To validate the model, we compared the treatment concordance rate with the National Comprehensive Cancer Network (NCCN) Guidelines, a representative set of cancer treatment guidelines, and with the results of the Gachon Gil Medical Center’s Colorectal Cancer Treatment Protocol (GCCTP).

**Results:**

For the C3R model, the treatment concordance rates with the NCCN guidelines were 70.5% for Top-1 Accuracy and 84% for Top-2 Accuracy. The treatment concordance rates with the GCCTP were 57.9% for Top-1 Accuracy and 77.8% for Top-2 Accuracy.

**Conclusions:**

This model is significant, i.e., it is the first colon cancer treatment clinical decision support system in Korea that reflects actual data. In the future, if sufficient data can be secured through cooperation among multiple organizations, more reliable results can be obtained.

## Background

Becoming a medical specialist generally requires 10–15 years of training, starting from entrance into university. A medical specialist determines the condition of a patient and makes an appropriate diagnosis based on medical and empirical knowledge acquired through years of experience. Nevertheless, many patients die every year from medical errors. According to a recent study performed at Johns Hopkins University, over 250,000 people in the United States died because of medical errors, which was the third leading cause of death after heart disease and cancer that year [1]. Medical errors also cost $20 billion annually in United States; minimizing medical errors is therefore crucial [2].

Clinical Decision Support Systems (CDSSs) have attracted attention as a method for minimizing medical errors [3]. CDSSs can help clinicians make rational decisions based on clinical information while diagnosing and treating diseases [4]. They can be applied to support decisions regarding prevention, diagnosis, treatment, prescription, and prognosis, but they are typically used for diagnosis and treatment. In terms of technology, CDSSs can be categorized as knowledge-based and non-knowledge-based [5]. Knowledge-based CDSSs provide rule-based decision making in accordance with a knowledge base of medical data generated in clinical environments. In contrast, non-knowledge-based CDSSs provide decision-making support by learning patterns in clinical medical information through artificial intelligence (AI) technologies, such as deep learning and machine learning. With the advancement of AI, significant developments are expected in non-knowledge-based CDSSs. However, the obstacle of securing data and verifying its integrity remains. In Korea in particular, it is difficult to use high-quality medical data freely under the Personal Information Protection Act [6].

Watson for Oncology (WfO)—a leading non-knowledge-based CDSS—was developed in 2012 by IBM through collaboration with the Memorial Sloan Kettering Cancer Center (MSKCC), which is the largest private hospital in New York [7]. WfO makes recommendations for diagnosing and treating cancer using models trained by internalizing medical big data, including 25,000 patient cases, 290 medical journals, and 12 million pages of specialized data [8, 9]. In a study published by the American Society of Clinical Oncology (ASCO) in 2014, WfO was used to evaluate the treatment of 200 leukemia patients with a concordance of 82.6% [10]. Moreover, a 2014 study by MSKCC indicated high treatment concordance rates for certain carcinomas, including colorectal cancer (98%) and cervical cancer (100%) [10]. However, according to data released in 2017 by the Gachon Gil Medical Center, which was the first hospital in Korea to introduce WfO, the diagnosis concordance rate has decreased for most cancers [11]. The diagnosis concordance rate for colorectal cancer was approximately 65.8%, a reduction of over 25% since WfO was first introduced [11]. This is because the NCCN guidelines, to which WfO refers for diagnosis of colorectal cancer, suggest only comprehensive treatment methods and do not consider individual patient characteristics. For this reason, Strickland [12] writes, “It was argued that WfO is difficult to use because it sometimes provides dangerous recommendations.” Furthermore, WfO is unable to link clinical medical data generated at the clinical site, e.g., electronic medical records (EMR). It has also been criticized in terms of its usability [13].

In many clinical fields, research is being conducted to establish decision support systems like WfO. In terms of knowledge-based CDSSs, Rocha et al. [14] proposed a shared-decision making-based CDSS for the treatment of prostate cancer. This model compares the results of WfO with the results of a shared-decision making process, which involves informed value-based selection with patients in the absence of a best treatment option. Perfect matches were found in 58%, partial matches in 15%, and inconsistencies in 31%. The main reason for inconsistencies was found to be that patients wanted treatment beyond surveillance. Krens et al. [15] established a CS rule-based CDSS for the treatment of kidney failure in cancer patients. Clinical rules were defined for a total of 18 cytotoxic drugs, and only 112 of the 2681 prescriptions generated warnings. A similar study presented a CDSS for the differential diagnosis of pulmonary fibrosis [16].

In the field of non-knowledge-based CDSSs, Pyo et al. [17] built a model to predict an anti-PD-1 cancer immunotherapy response using clinical and blood-based data from lung cancer patients. Supervised machine learning models such as LASSO, Ridge Regression, Elastic Net, Support Vector Machine (SVM), Artifical Neural Network (ANN), and Random Forest (RF) were used. Among them, the ridge regression model (area under the ROC curve (AUC): 0.78) showed excellent performance in predicting the anti-PD-1 response. Kenny et al. [18] proposed a CDSS based on computed tomography (CT) to evaluate the response to muscle-invasive bladder cancer treatment. To confirm the degree of response before and after chemotherapy, they constructed CDSS-T, a deep learning model based on a convolutional neural network, using CT images and radioactive features. The mean AUC value for CDSS-T was 0.80, compared to an AUC value of 0.74 for doctors who did not use CDSS-T. Although various studies have been conducted to establish CDSSs, most involve rule-based CDSSs that do not reflect real-world data or CDSSs that simply predict the onset. According to the 2016 National Cancer Registration Statistics released by the Central Registration Center in 2018, colorectal cancer is the second most common type of cancer (after gastric cancer) and is the fourth most common type of cancer in the United States [19, 20]. Moreover, because the recurrence rate (i.e., recurrence of the primary cancer or a new cancer) after the treatment of colorectal cancer is higher than for other carcinomas, it is important to select an appropriate chemotherapy treatment recommendation.

Therefore, to resolve the limitation that existing non-knowledge-based CDSSs do not reflect actual data, we developed an EMR data-based deep learning model called the Colorectal Cancer Chemotherapy Recommender (C3R). Oversampling was used to solve the problem of overfitting caused by the class imbalance, which led to a significant improvement in performance. In addition, the Deep-Surv model was used to support more accurate decision making by checking which factors influenced the chemotherapy recommendation in the deep learning process.

The structure of the manuscript is as follows. In the Background section, we briefly introduce clinical decision support systems. In the Methods section, we present our contributions, including our implementation of data extraction and data preprocessing and our proposed treatment recommendation model C3R. In the Results section, we describe the experimental design and results. The Conclusion and Discussion sections conclude the manuscript.

## Methods

### Dataset

In this study, we used the EMR data of the Gachon Gil Medical Center, which launched WfO of IBM in Korea for the first time in 2016. The Gachon Gil Medical Center obtained reliable data through WfO and multi-disciplinary medical treatments involving face-to-face interaction between patients and four or more cancer treatment specialists. The data were collected from patients who had undergone colorectal cancer surgery between 2004 and 2012. The dataset includes information such as demographics and disease, cancer, tumor, treatment, survival, and genetic characteristics. This standard information is based on the colorectal cancer Common Data Model (CDM) definition employed by five domestic hospitals, including the Gachon Gil Medical Center.

The EMR data of the Gachon Gil Medical Center are divided into scanned, XML, and database EMRs according to the storage method. In scanned and XML EMRs, it is possible that the data were deleted or entered incorrectly when a medical record administrator checked the record. Therefore, to verify the reliability and integrity of the extracted dataset, several colon cancer specialists and medical record administrators collaborated to review the charts.

The chart review involved a detailed three-step process over a six-month period. In the first step, the extracted data were checked to ensure that they were properly mapped with the code described in the colorectal cancer CDM definition document and were then extracted from the correct location through the normal method. In the second step, to ensure the reliability of the extracted data, an operation was performed to identify and remove incorrect data, such as redundancies and incorrect inputs. This chart review process was repeated at monthly intervals under the supervision of a colorectal cancer specialist. In the final step, to reduce unnecessary biases in the training of the deep learning model, the colorectal cancer specialists selected first-priority variables that are highly related to survival. Table 1 presents six data categories and the variables in each category.

**Table 1:** Dataset description

Input Variables	
Demographics	Age, Sex, ASA, BMI, Smoking History
Disease Characteristics	DM History, Pulmonary Disease, Liver Disease, Heart Disease, Kidney Disease
Cancer Characteristics	Prior Cancer Diagnosis, Initial CEA, Perforation, Obstruction, Emergency, Lymphovascular Invasion, Perineural Invasion, Distal Resection Margin, Radial Margin, Radiotherapy, Harvested Lymph Node, Positive Lymph Node, Early Complication
Tumor Characteristics	Hereditary Colorectal Tumor, Tumor Location (Pathology), Histologic Type, TNM Stage (Pathology)
Genetic Characteristics	K-ras, N-ras, BRAF
Treatment Characteristics	Postoperative Chemotherapy
Oncologic Outcomes	Overall Survival, Recurrence
**Target Variables**	
Chemotherapy	Postoperative Chemotherapy Regimen (5-FU/LV, XELODA, FOLFOX, FOLFIRI, Surveillance)

### Data Preprocessing and oversampling

#### Data Preprocessing

Data preprocessing is often required to obtain correct analysis results. If data preprocessing is not performed correctly, the relationship between the variables may be distorted [21]. Data preprocessing is therefore important for generating a solid model. In this study, we focused on pre-processing missing values and on categorical and continuous variables prior to constructing the deep learning models.

**Fig. 1:**
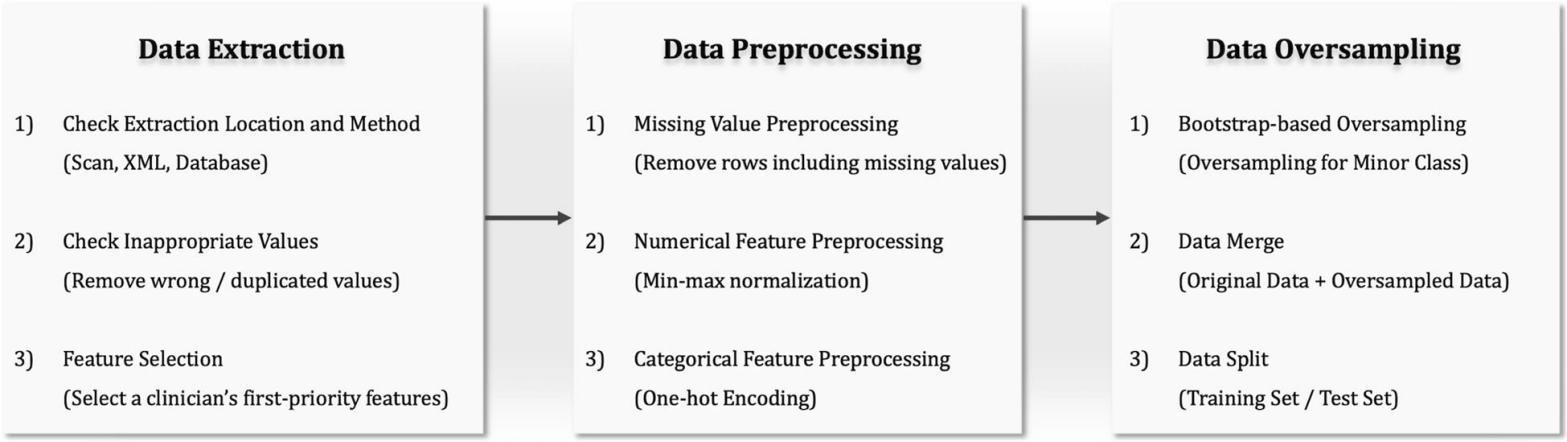
Data preprocessing and data oversampling

First, if the missing-value ratio of a variable was determined to be > 90%, the variable was excluded because sufficient data samples for training could not be obtained. All instances of missing values in the prediction target class Post-OP Chemo Regimen were excluded. Continuous variables such as age, ASA, and CEA have different ranges, and if training is performed without adjusting the ranges, overfitting may occur, obstructing normal learning [22]. Therefore, the range of each variable was scaled to − 1 to 1 by applying the min-max normalization scaling method. In the case of the categorical variables, the values were mostly character data rather than numeric data, and thus could not be automatically recognized or computed by a computer. One-hot encoding was therefore employed to vectorize each variable and represent it as 0 s and 1 s. Figure 1 illustrates the data preprocessing process, which includes a data oversampling process.

### Data oversampling

**Fig. 2:**
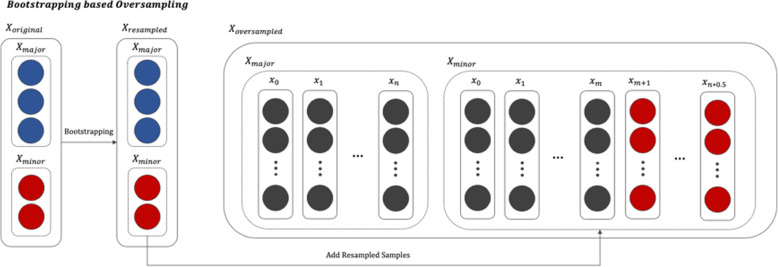
Process of the bootstrap-based oversampling algorithm (blue nodes represent the majority class, and red nodes represent the minority class of the target variable. Oversampling is limited to the minority class, i.e., the oversampled data is only added to the minority class)

In most real world data, the classes of the target variables have an imbalanced distribution [23, 24]. Data that have such a distribution are called imbalanced data. Medical data generated in a clinical environment are particularly severely imbalanced. Normally, we define a class with a relatively small proportion of the total instances as a minor class and a class with a large proportion of instances as a major class [25]. If model training is performed using imbalanced data, it is likely that the minor class will not be properly recognized, and all test data will be classified as belonging to the major class [26]. Various methods, such as undersampling and oversampling, have been proposed to solve this problem. Undersampling involves adjusting the class proportions by removing some data from the major class, whereas oversampling involves reproportioning the classes by multiplying the minor class data. In general, when there is sufficient data, undersampling is used. However, undersampling would hinder the construction of a normal learning model in this study because the dataset is not sufficiently described. We therefore attempted to resolve the data imbalance by oversampling using the bootstrap resampling algorithm [27], which allows for effective inference with a small amount of data. The oversampled data is only added to the minority class in the training set to avoid affecting the test performance. Figure 2 shows a bootstrap-based oversampling process.

### Structure of the chemotherapy recommender

To predict and recommend treatment methods, we developed a deep feed-forward neural network, called the Colorectal Cancer Chemotherapy Recommender (C3R). It is the most basic implementation of a deep neural network (DNN). The model was designed as a three-layer perceptron structure in the order of [Input Layer] – [Hidden Layer] – [Output Layer]. The detailed nodes composing each layer were designed as ([Input: 54] – [Hidden: 64] – [Hidden: 128] – [Hidden: 256] – [Hidden: 64] – [Output: 5]). We used a grid-search algorithm to tune the hyperparameters. The hyperparameter types and grid-search ranges were as follows: layers ∈ {1, 2, 3, 4}, batch size ∈ {32, 64, 128, 256}, learning rate ∈ {0.1, 0.01, 0.001, 0.0001}, and optimization algorithm ∈ {Adam [28], Adadelta [29], RMSProp [30]}. The hyperparameters determined by the grid-search algorithm were [Batch Size 64, Learning Rate 0.001, and Optimization Algorithm: Adam Optimizer]. A dropout layer was added in the middle of each hidden layer to prevent overfitting. ReLU [31] was used as the activation function for each layer except the output layer for which Softmax [32] was used as the activation function. The Softmax activation function calculates the input data and returns a probability value normalized between 0 and 1; it can be expressed as follows:

1 $$ \sigma {\left(\boldsymbol{z}\right)}_i=\frac{e^{z_i}}{\sum_{j=1}^K{e}^{\beta {z}_i}}\  for\ i=1,\dots, K\  and\ z=\left({z}_1,\dots, {z}_K\right)\in {\mathbb{R}}^K $$

**Fig. 3:**
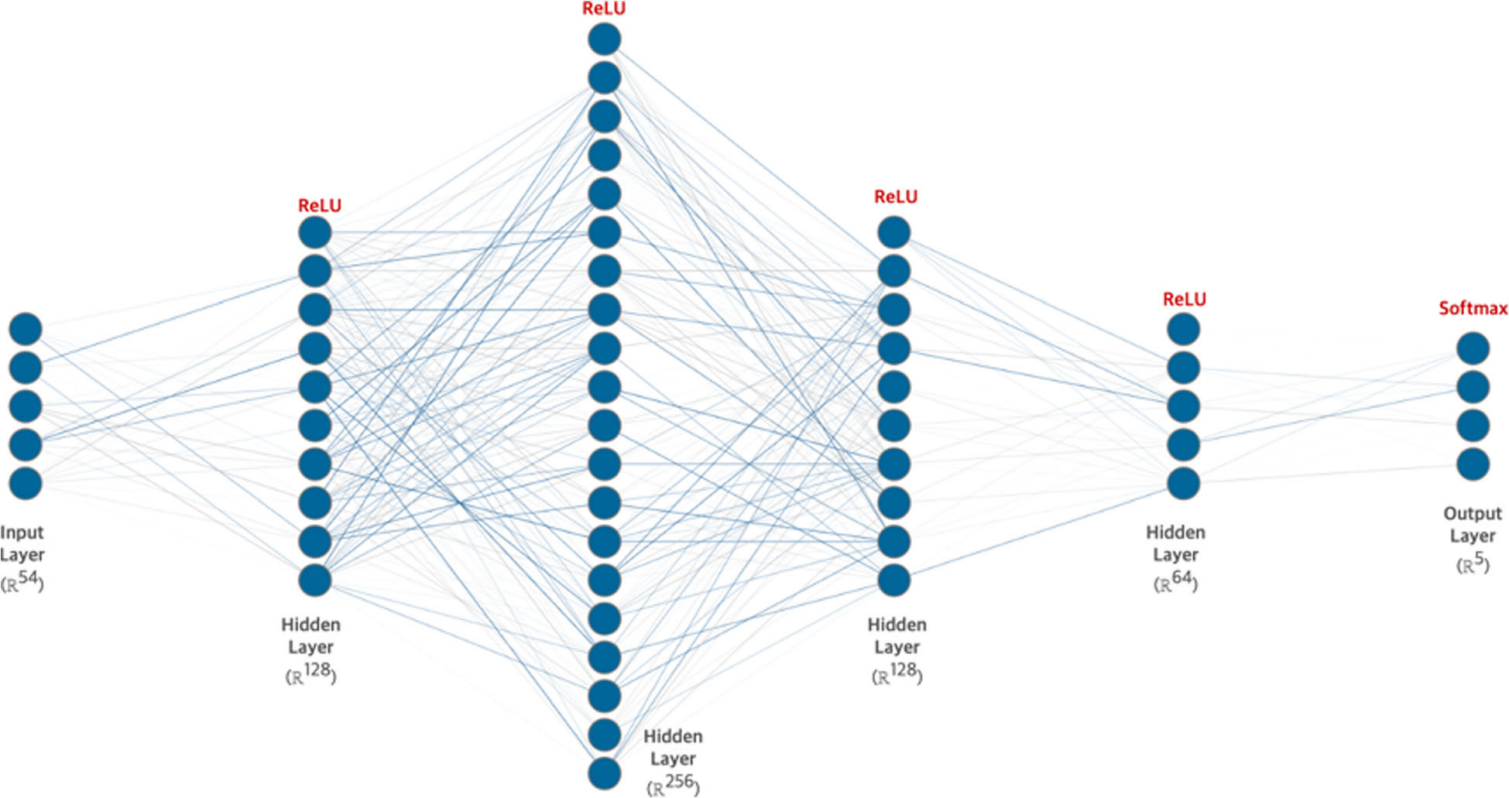
Structure of the deep learning model for chemotherapy recommendation

The returned probability value is defined as the Chemotherapy Recommendation Index, and according to this value, a priority can be determined for suggesting an appropriate treatment method to the patient. Figure 3 illustrates the detailed structure of C3R.

### Model verification and evaluation

**Fig. 4:**
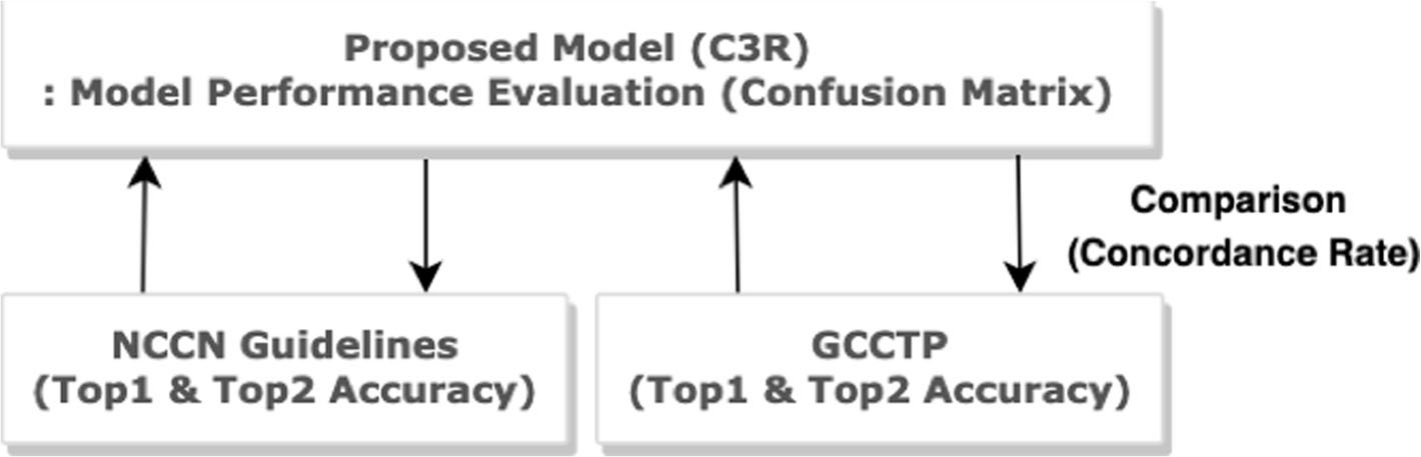
Process of model evaluation and verification

To evaluate the performance of the proposed C3R model, we used a confusion matrix. We then compared the diagnosis concordance rate between the C3R model and the Gachon Colorectal Cancer Treatment Protocol (GCCTP) and NCCN guidelines to validate C3R. Top-1 Accuracy and Top-2 Accuracy were used as comparative indicators because the treatment methods proposed in each guideline were broken down by priority. The recommendations of the C3R model are considered to have Top-1 Accuracy if they are included in the preferred treatment method proposed by each guideline, and they are considered to have Top-2 Accuracy if they are included in the next suggested treatment. Figure 4 shows the model verification process including the model performance evaluation.

### Gachon colorectal Cancer treatment protocol

**Fig. 5:**
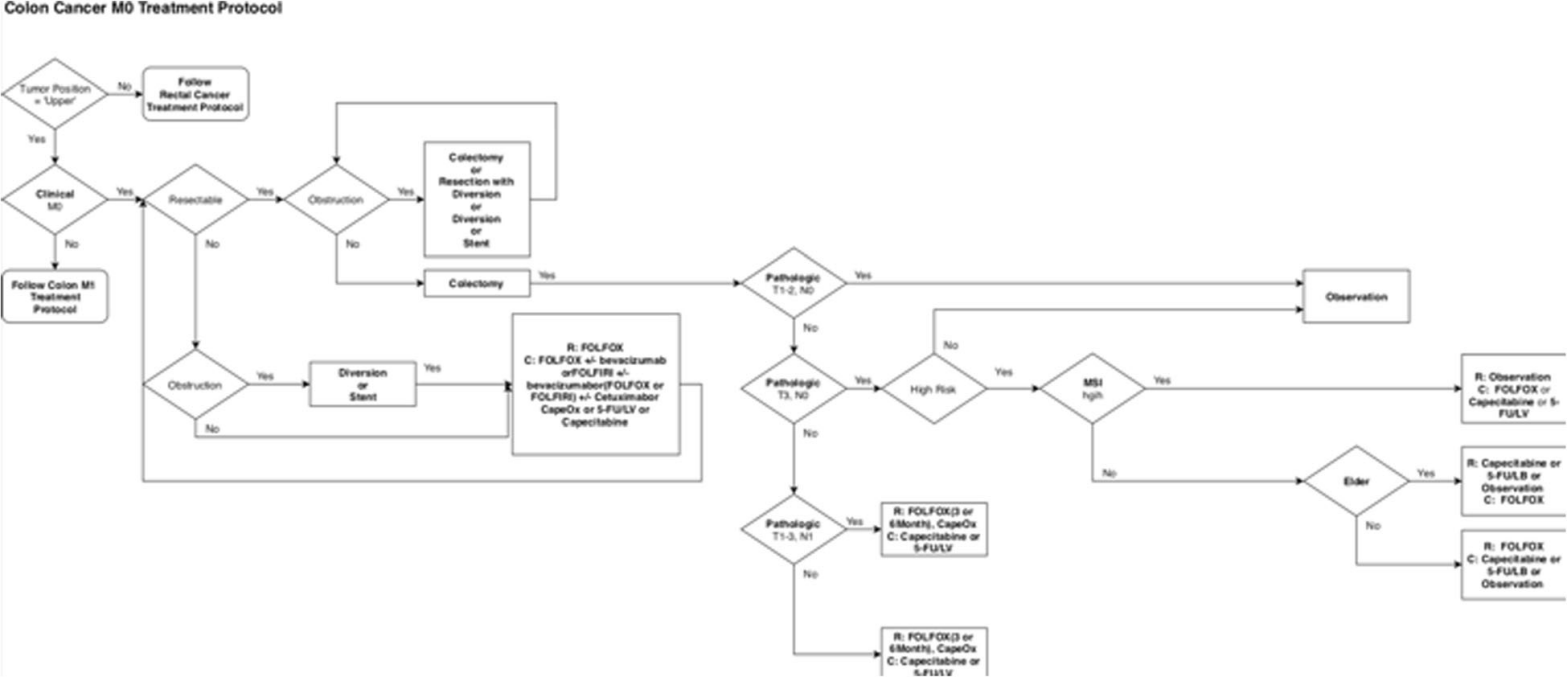
Example of a CRC treatment protocol: Colon Cancer M0 Treatment Protocol (the protocol is an algorithm that is used when administering chemotherapy to patients with colorectal cancer at the Gachon Gil Medical Center. The protocol recommendations are generally divided for rectal cancer and colon cancer and for M0 and M1 cases without metastasis)

For validation, we first used the GCCTP, which colorectal cancer specialists use to determine treatment options for patients at the Gachon Gil Medical Center. The GCCTP is a rule-based treatment recommendation system based on empirical knowledge from numerous colorectal cancer specialists. It allows a colorectal cancer specialist to diagnose a patient’s condition and determine treatment options according to information such as the patient’s demographics, TNM stage, and risk factors. Figure 5 shows an example of a colorectal cancer treatment protocol based on the GCCTP for a case without metastasis.

### NCCN guidelines

The NCCN guidelines, which were published by experts from 28 cancer centers in the United States, reflect the opinions of experts and serve as a guideline for international cancer treatment standards. The guidelines cover the diagnosis, treatment decisions, and treatments for 97% of the cancers in the United States. They are updated annually with new medical grounds to provide the optimal clinical guidelines for treating cancer patients. The NCCN guidelines are divided into rectal and colon cancer guidelines. Version 2 of 2019 was used for verification of colon cancer treatment recommendations and Version 2 of 2018 for verification of rectal cancer treatment recommendations [33, 34].

### Performance evaluation metrics

Various comparative indicators were used to evaluate the performance of the C3R model. Specifically, we used a confusion matrix to evaluate the model performance. A confusion matrix, which is typically used to evaluate the performance of an algorithm [35], compares the actual results with the model prediction results in a table that includes four categories: true positive (TP), true negative (TN), false positive (FP), and false negative (FN). TP refers to a true prediction when the actual result was true and TN to a false prediction when the actual result was false. FP refers to a false prediction when the actual result was true and FN to a negative prediction when the actual result was true. These metrics can be used to calculate evaluation indicators, such as the accuracy, sensitivity, specificity, precision, recall, F1-Score, and area under the ROC curve (AUC) [36]. In this study, we used the precision, recall, F1-score, and AUC, which can be used regardless of class imbalances.

## Results

### Results of data extraction and data Preprocessing

The initial EMR dataset consisted of 143 variables and 1511 instances. After the chart review and data preprocessing, the dataset consisted of 59 variables and 1169 instances. Of the 59 variables, 5 were selected as target classes and were ultimately predicted and recommended: 5-FU/LV, XELODA, FOLFOX, FOLFIRI, and Surveillance. We divided the final dataset into a training set for learning and a test set for model verification (at a ratio of 8:2), to construct a deep learning model. Table 2 provides the detailed process of chart review and data preprocessing. Details of the continuous and categorical variables can be found in the S1 and S2 Tables, respectively.

**Table 2:** Dataset changes due to chart review and data preprocessing

Process		Variables (+Target Classes)	Patients (*N*)
First CRC Dataset		142 (+ 1)	1511
Chart Review	1) Check extraction method and location	142 (+ 1)	1508
	2) Check for inappropriate data	142 (+ 1)	1496
	3) Select priority variables (First Processed CRC Dataset)	40 (+ 1)	1496
Data Preprocessing	1) Drop redundant variables	37 (+ 1)	1496
	2) Drop variables including 90% ↑ missing values	32 (+ 1)	1496
	3) Drop instances containing missing values	32 (+ 1)	1169
	4) One-hot encoding (Final CRC Dataset)	54 (+ 5)	1169
Data Split	1) Data split (training/testing)	54 (+ 5)	935 / 234

### Results of data oversampling

Through the bootstrap resampling process, we created sufficient minor class data (XELODA and FOLFIRI) for training. The minor class data was oversampled from the existing data by a factor of approximately 5, and the major class was not oversampled. The newly created data were only added to the training set to avoid affecting the test results. The numbers of instances in each target class after the oversampling are presented in Table 3.

**Table 3:** Results of oversampling of the minor classes

Method	Total	5-FU/LV	XELODA	FOLFOX	FOLFIRI	Surveillance
Original	1169	398	42	323	35	371
After Oversampling	**1454**	398	**206**	323	**156**	371

To check whether the distribution of the generated data resembled that of the existing data, the mean and standard deviation of Age and OS were calculated for each anticancer treatment method according to gender. A t-test was conducted to check whether there was a difference between the two groups for XELODA and FOLFIRI, for which data oversampling was performed (Table 3). The mean age of the XELODA male group was 68.00 years, and the standard deviation was 9.90 years, indicating no significant difference (95% confidence interval: 63.90–72.10 years). The mean OS of the FOFIRI female group was 35.04, and the standard deviation was 33.64. As for these two examples, none of the indicators of XELODA and FOLFIRI exhibited a significant difference in mean after oversampling (*p* > 0.05). Details of the t-test can be found in S3 Table. This result indicates that the data generated using the bootstrap resampling technique had a similar distribution to the existing data. The newly generated data were thus added to the existing minor class to enable effective learning of the minor class.

### Performance of C3R

**Fig. 6:**
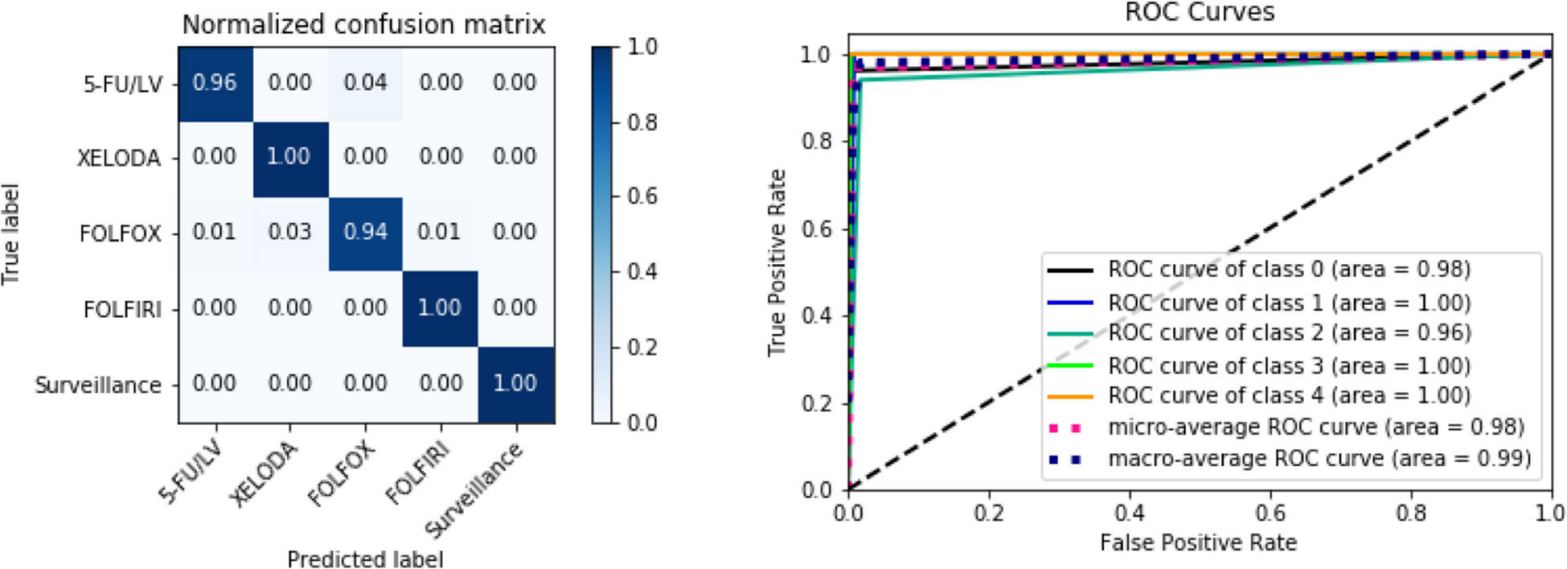
ROC curve and confusion matrix for evaluation of the proposed model

As mentioned above, we used the precision, recall, F1-score, and AUC indices to evaluate the performance of the proposed C3R anticancer treatment recommendation model. The AUC values for all classes were > 0.95, indicating that the developed model generally had good performance. For surveillance, all patient cases were predicted correctly, i.e., with 100% accuracy. For the oversampled variables, i.e., XELODA and FOLFIRI, the precision values were 0.80 and 0.89, respectively. The overall performance of the model on all classes was generally good, with a precision of 0.92, recall of 0.98, F1-score of 0.95, and AUC of 0.98 (Table 4, Fig. 6).

**Table 4:** Performance of the proposed model for each chemotherapy method

Class	Precision	Recall	F1-score	AUC
5-FU/LV	0.99	0.96	0.97	0.97
XELODA	0.80	1.00	0.89	0.99
FOLFOX	0.95	0.94	0.95	0.96
FOLFIRI	0.89	1.00	0.94	0.99
Surveillance	1.00	1.00	1.00	1.00
Total	0.92	0.98	0.95	0.98

To evaluate the performance of C3R objectively, we also compared its performance with that of other machine learning algorithms such as SVM, Decision Tree, K-NN, and RF. The results indicate that the proposed model performed best followed by the Decision Tree. This may have occurred because the Decision Tree is effective algorithm for classifying binary data. Table 5 compares the performance of the algorithms.

**Table 5:** Comparison of the performance of the proposed model and various machine learning algorithms

Method	Precision	Recall	F1-score	AUC
Proposed	**0.92**	**0.98**	**0.95**	**0.98**
SVM	0.80	0.90	0.89	0.85
Decision Tree	0.90	0.94	0.93	0.93
K-NN	0.82	0.83	0.80	0.82
Random Forest	0.91	0.93	0.92	0.92

### Model verification

To verify C3R, we randomly extracted 200 data instances from a test set that was not involved in model training. Of these instances 24 were excluded from the comparison evaluation because the GCCTP treatment protocol did not reflect the variables used in the C3R model. Table 6 presents a comparison of the chemotherapy treatment methods recommended by C3R and those recommended by GCCTP and NCCN. XELODA and FOLFIRI, for which there were insufficient test samples, exhibited fluctuations in Top-1 and Top-2 Accuracy.

**Table 6:** Comparison of the Top-1 and Top-2 Accuracy between the proposed model and the GCCTP and NCCN guidelines

*Group*		*N*	Top-1 Accuracy (%)	Top-2 Accuracy (%)
GCCTP	5-FU/LV	55	23.63	78.18
	XELODA	5	80.00	80.00
	FOLFOX	37	83.78	91.89
	FOLFIRI	4	0	75.00
	Surveillance	76	71.05	71.05
	**Total**	**176**	**57.95**	**77.84**
NCCN	5-FU/LV	59	47.45	83.05
	XELODA	6	50.00	100.00
	FOLFOX	50	92.00	94.00
	FOLFIRI	5	100.00	100.00
	Surveillance	80	80.00	80.00
	**Total**	**200**	**70.50**	**84.00**

The GCCTP treatment concordance rate was 57.95% for Top-1 Accuracy and 77.84% for Top-2 Accuracy. For cases of 5-FU/LV and FOLFIRI, the Top-1 Accuracy was 23.63 and 0%, respectively. For XELODA, FOLFOX, and Surveillance, however, the Top-1 Accuracy ranged from 70 to 80%. The Top-2 Accuracy for FOLFOX was 91.89%, which was the highest treatment concordance rate among the chemotherapy methods.

The treatment concordance rate with the NCCN guidelines was higher than for the GCCTP, with a Top-1 Accuracy of 70.50% and a Top-2 Accuracy of 84%. Except for 5-FU/LV and XELODA, a treatment concordance rate of > 80% was achieved for all chemotherapy treatment methods. Although FOLFIRI was limited to five samples, both the Top-1 and Top-2 Accuracy achieved a 100% treatment concordance rate.

### Model explanation

To explain why the C3R model recommends specific treatment options, we use the SHapley Additive exPlanations (SHAP) model. The SHAP model [37] is a game theoretic approach to explaining the output of a machine learning model. It connects the optimal credit allocation with local explanations using the classic Shapley values from game theory and their related extensions.

**Fig. 7:**
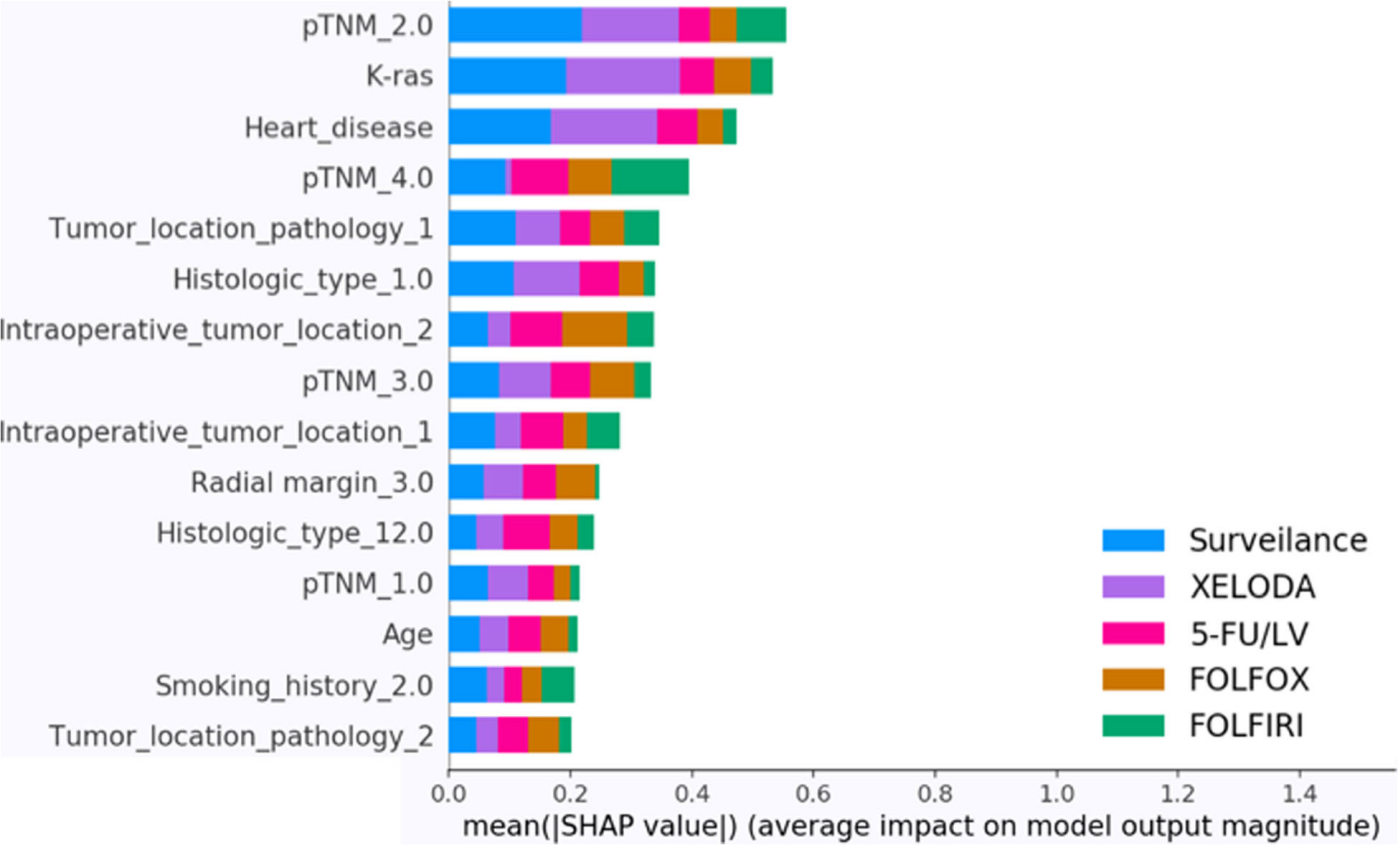
Overview of the most important variables to the model

Figure 7 illustrates the contribution of different variables to the model output. In general, pathologic variables such as the TNM Stage and tumor location were found to have a significant effect on the model. In addition, demographics such as age, smoking history, and histologic type were also found to influence the results.

## Discussion

In this study, we propose a DNN-based deep learning model called C3R to provide chemotherapy recommendations for colorectal cancer patients after surgery. One limitation in this study is that the model was built using specific data from a single institution. It can be assumed that this data is generalized to match the data collected at certain hospitals. To minimize this generalization problem and to ensure the scalability of the model developed in this study, we extracted data based on the Colorectal Cancer Data Dictionary that was built through collaboration with various domestic hospitals. The Colorectal Cancer Data Dictionary is a Common Data Model (CDM) for colorectal cancer and was created to unify data variables and formats occurring across hospitals. It is currently constructed using the data from a single hospital, but in the future, it can accommodate data collected from various hospitals. To expand the model proposed in this study, a test will be conducted for patients with colorectal cancer at the Gachon Gil Medical Center.

The treatment concordance rates of the C3R model with the NCCN guidelines were 70.5% for Top-1 Accuracy and 84% for Top-2 Accuracy. This is approximately 10% greater than the rates for the GCCTP; the special medical insurance system of Korea makes it difficult to use the chemotherapy methods recommended by the NCCN. In Korea, chemotherapy is proposed to patients on the basis of guidelines provided by the Health Insurance Review and Assessment Service (HIRA). If a patient selects a treatment that does not satisfy the guidelines of the HIRA, they are required to pay a substantial fee because they will not be eligible for health insurance. Most patients therefore select chemotherapy methods that satisfy the HIRA guidelines. This may have reduced the diagnosis concordance rate in the present study because the treatment methods proposed by the C3R model and the NCCN guidelines may differ.

Katzman JL et al. [38] proposed DeepSurv, which was briefly introduced in the Model Explanation section for the identification of variables affecting the model. DeepSurv is a deep learning-based recommendation model based on patient survival data. The DeepSurv model requires accurate tracking to determine a patient’s prognosis after chemotherapy. To determine the exact prognosis for a patient, continuous follow-up, typically over 3 to 5 years, is required. Realistically, it is not easy to follow a patient for such a long duration. Another limitation is that if a patient dies within the follow-up period, it can be difficult to determine the exact cause of death because several factors may be involved. Our proposed method differs in that the developed model recommends chemotherapy using objective data that can be extracted from patients.

To expand the model proposed in this study, tests will be conducted on colon cancer patients visiting the Gachon Gil Medical Center. During the test, we will analyze the match rate of the chemotherapy recommendations with clinicians to confirm that we are properly supporting decision making. The model performance can then be further enhanced through a series of processes that will expand the model to multiple connected hospitals to collect refined data. With more research at scale, CR3 can be used by clinicians to select personalized treatment options.

## Conclusions

The C3R model is a CDSS based on data generated at the Gachon Gil Medical Center. It learns past clinical cases to recommend personalized treatment methods. The AUC of the C3R model was approximately 0.98, indicating excellent performance on the EMR data, but the treatment concordance rates with the GCCTP and NCCN guidelines were not as high. The GCCTP—a treatment protocol established through the cooperation of several colorectal cancer specialists—is limited in that not all the data generated in the clinical environment are reflected in the rule-based system. While over 40 variables are used in the C3R model, approximately 20 variables were used to construct the GCCTP.

The CR3 reflects actual data, in contrast to existing non-knowledge-based CDSSs. Its development is significant, i.e., it is the first colon cancer treatment method decision support system in Korea that reflects actual data. From a clinical viewpoint, if a CDSS built with a vast amount of data outputs results that differ from existing treatment protocols and guidelines, the results may be accepted as a new opinion for the treatment protocol rather than treated as an algorithmic error. Moreover, an interpretable CDSS can be built by providing deep learning models and a deep learning interpretation model.

## Supplementary information

**Additional file 1 Table S1** Patient characteristics (numeric variables). **Table S2** Patient characteristics (categorical variables). **Table S3.** Comparison of descriptive statistics before and after oversampling, based on T-tests.

## Data Availability

The data are available from the Gachon Gil Medical Center but restictions apply to the availability of these data. The data were used under license for current study, and so are not publicly available. The data are, however, available from the authors upon reasonable request and with the permission of the Gachon Gil Medical Center.

## Abbreviations

AI: Artificial intelligence
ANN: Artificial Neural Network
ASCO: American Society of Clinical Oncology
AUC: Area under the curve
CDM: Common data model
CDSS: Clinical decision support system
CT: Computed tomography
C3R: Colorectal cancer chemotherapy recommender
DNN: Deep neural network
EMR: Electronic medical records
FN: False negative
FP: False positive
GCCTP: Gachon Colorectal Cancer Treatment Protocol
HIRA: Health insurance review and assessment service
MSKCC: Memorial Sloan Kettering Cancer Center
NCCN: National Comprehensive Cancer Network
RF: Random Forest
SHAP: Shapley additive explanations
SVM: Support Vector Machine
TN: True negative
TP: True positive
WfO: Watson for oncology
