# Supplementary material for: A data-driven approach to a chemotherapy recommendation model based on deep learning for patients with colorectal cancer in Korea
Source: BMC Med Inform Decis Mak. 2020 Sep 22;20:241. doi: 10.1186/s12911-020-01265-0 (PMC7510149; doi:10.1186/s12911-020-01265-0)
Supplement: Supplementary file 1 — Additional file 1 Table S1 Patient characteristics (numeric variables). Table S2 Patient characteristics (categorical variables). Table S3. Comparison of descriptive statistics before and after oversampling, based on T-tests. [file 12911_2020_1265_MOESM1_ESM.docx]

**Supporting Information**

**S1 Table.** Patient characteristics (numeric variables).

| Variable | Number of Samples | Mean / SD |
| --- | --- | --- |
| Age | 1169 | 63.14 / 11.57 |
| BMI |  | 23.14 / 3.25 |
| Initial CEA |  | 12.27 / 60.86 |
| Harvest Lymph Node |  | 22.16 / 16.39 |
| Positive Lymph Node |  | 2.39 / 5.49 |
| OS |  | 77.58 / 40.77 |

**S2 Table.** Patient characteristics (categorical variables)

| Variables | | *N* | Variables | | *N* | Variables | *N* | |
| --- | --- | --- | --- | --- | --- | --- | --- | --- |
| Total | 1169 | | **Related Disease** | | | **Cancer Characteristics** | | |
| **Demographics** | | | Diabetes |  | | Prior Cancer Diagnosis | | |
| Sex |  | | No | 992 | | No | | 1126 |
| Male | 709 | | Yes | 177 | | Yes | | 26 |
| Female | 460 | | Pulmonary |  | | No Information | | 17 |
| ASA |  | | No | 1101 | | Perforation | |  |
| 1 | 128 | | Yes | 68 | | No | | 955 |
| 2 | 952 | | Liver |  | | Yes | | 214 |
| 3 | 88 | | No | 1146 | | Obstruction | |  |
| 4 | 1 | | Yes | 23 | | No | | 1131 |
| Smoking History | | | Heart |  | | Yes | | 38 |
| No | 473 | | No | 803 | | Emergency | |  |
| Yes | 71 | | Yes | 366 | | No | | 1140 |
| No Information | 625 | | Kidney |  | | Yes | | 29 |
| **Genetic Characteristics** | | | No | 1161 | | Lymphovascular Invasion | | |
| K-ras |  | | Yes | 8 | | Lymphatic | | 255 |
| Not Assessed | 923 | | **Tumor Characteristics** | | | Vascular | | 16 |
| Wild Type | 246 | | Hereditary CRC Tumor | | | Lymphovascular | | 883 |
| N-ras |  | | No | 1155 | | Not Assessed | | 15 |
| Not Assessed | 1169 | | FAP | 3 | | Perineural Invasion | | |
| BRAF |  | | HNPCC | 11 | | Present | | 162 |
| Not Assessed | 1169 | | Tumor Location |  | | Unidentified | | 646 |
| **Chemotherapy** | | | Colon | 645 | | Not Assessed | | 361 |
| Postoperative Chemotherapy Regimen | | | Rectum | 524 | | Distal Resection Margin | | |
| 5-FU/LV | 398 | | Histologic Type |  | | Not Involved | | 1085 |
| XELODA | 42 | | Adenocarcinoma | 527 | | Involved | | 6 |
| FOLFOX | 323 | | Well-Diff. | 84 | | Undescribed | | 78 |
| FOLFIRI | 35 | | Mod-Diff. | 528 | | Radial Margin | |  |
| Surveillance | 371 | | Poorly-Diff. | 30 | | Not Involved | | 462 |
| Postoperative Chemotherapy | | | TNM Stage (Pathology) | | | Involved | | 51 |
| No | 375 | | 0 | 13 | | Undescribed | | 656 |
| Yes | 794 | | 1 | 236 | | Early complication | |  |
| Recurrence |  | | 2 | 382 | | No | | 1140 |
| No | 1009 | | 3 | 423 | | Yes | | 29 |
| Yes | 160 | | 4 | 115 | | No information | | 0 |

**S3 Table.** Comparison of descriptive statistics before and after oversampling, based on T-tests.

| Before  Oversampling | Male | | | | | | Female | | | | | | | | | | Total | | | | | | | | | | | |
| --- | --- | --- | --- | --- | --- | --- | --- | --- | --- | --- | --- | --- | --- | --- | --- | --- | --- | --- | --- | --- | --- | --- | --- | --- | --- | --- | --- | --- |
|  | *N* | Age (mean/SD) | | | OS (mean/SD) | | | *N* | | Age (mean/SD) | | | | OS (mean/SD) | | | *N* | | | Age (mean/SD) | | | | | | OS (mean/SD) | | |
| 5-FU/LV | 144 | 62.53±12.10 | | | 93.50±42.09 | | | 254 | | 60.64±10.34 | | | | 89.54±40.62 | | | 398 | | | 61.32±11.03 | | | | | | 90.97±41.15 | | |
| XELODA | 22 | 68.00±9.90 | | | 70.72±44.76 | | | 20 | | 70.65±10.30 | | | | 59.20±36.39 | | | 42 | | | 69.26±10.06 | | | | | | 65.23±40.91 | | |
| FOLFOX | 125 | 59.60±11.15 | | | 77.81±37.83 | | | 198 | | 60.78±9.79 | | | | 73.34±35.60 | | | 323 | | | 60.32±10.33 | | | | | | 75.07±36.49 | | |
| FOLFIRI | 11 | 54.00±13.80 | | | 38.81±37.48 | | | 24 | | 63.95±7.97 | | | | 35.04±33.64 | | | 35 | | | 60.82±11.00 | | | | | | 36.22±34.38 | | |
| Surveillance | 158 | 67.69±12.81 | | | 73.62±42.11 | | | 213 | | 66.60±11.56 | | | | 68.53±37.23 | | | 371 | | | 67.06±12.10 | | | | | | 70.69±39.41 | | |
| Total | 709 | 62.86±10.89 | | | 76.00±39.74 | | | 460 | | 63.56±12.55 | | | | 80.01±42.25 | | | 1169 | | | 63.14±11.57 | | | | | | 77.58±40.77 | | |
| After  Oversampling | Male | | | | | | Female | | | | | | | | | | Total | | | | | | | | | | | |
|  | *N* | Age (mean/SD) | | | OS (mean/SD) | | | *N* | | Age (mean/SD) | | | | OS (mean/SD) | | | *N* | | | Age (mean/SD) | | | | | | OS (mean/SD) | | |
| 5-FU/LV | Same as Above | | | | | | | | | | | | | | | | | | | | | | | | | | | |
| XELODA | 111 | 67.34±11.79 | | | 67.14±45.42 | | | 95 | | 70.04±10.17 | | | | 62.06±33.11 | | | 206 | | | 68.60±11.13 | | | | | | 64.77±40.15 | | |
| FOLFOX | Same as Above | | | | | | | | | | | | | | | | | | | | | | | | | | | |
| FOLFIRI | 38 | 51.88±13.28 | | | 40.56±34.78 | | | 118 | | 63.07±7.76 | | | | 38.93±36.54 | | | 156 | | | 61.13±11.43 | | | | | | 39.42±35.95 | | |
| Surveillance | Same as Above | | | | | | | | | | | | | | | | | | | | | | | | | | | |
| Total | 709 | 62.86±10.89 | | | 76.00±39.74 | | | 460 | | 63.56±12.55 | | | | 80.01±42.25 | | | 1169 | | | 63.14±11.57 | | | | | | 77.58±40.77 | | |
| 2-sample  T-test | Male | | | | | | | | Female | | | | | | | | | | Total | | | | | | | | | |
|  | Age | | | OS | | | | | Age | | | | OS | | | | | | Age | | | | | | OS | | | |
|  | t | | p | t | | p | | | t | | p | | t | | | p | | | t | | | p | | t | | | | p |
| XELODA | 0.276 | | 0.784 | 0.341 | | 0.734 | | | 0.241 | | 0.811 | -0.324 | | | | 0.748 | | | 0.380 | | | 0.705 | | 0.066 | | | | 0.947 |
| FOLFIRI | 0.469 | | 0.645 | -0.138 | | 0.891 | | | 0.495 | | 0.623 | -0.508 | | | 0.614 | | | -0.149 | | | 0.881 | | -0.493 | | | | 0.623 | |
